# Identification of functional and diverse circulating cancer‐associated fibroblasts in metastatic castration‐naïve prostate cancer patients

**DOI:** 10.1002/1878-0261.13653

**Published:** 2024-04-17

**Authors:** Richell Booijink, Leon W. M. M. Terstappen, Eshwari Dathathri, Khrystany Isebia, Jaco Kraan, John Martens, Ruchi Bansal

**Affiliations:** ^1^ Personalized Diagnostics and Therapeutics, Department of Bioengineering Technologies, Technical Medical Centre, Faculty of Science and Technology University of Twente Enschede The Netherlands; ^2^ Department of Medical Cell BioPhysics, Technical Medical Centre, Faculty of Science and Technology University of Twente Enschede The Netherlands; ^3^ Department of General, Visceral and Pediatric Surgery University Hospital Düsseldorf, Heinrich‐Heine University Germany; ^4^ Department of Medical Oncology Erasmus MC Cancer Institute, University Medical Center Rotterdam The Netherlands

**Keywords:** circulating cancer‐associated fibroblast, circulating tumor cells, fibroblast‐activated protein, liquid biopsy, metastatic prostate cancer

## Abstract

In prostate cancer (PCa), cancer‐associated fibroblasts (CAFs) promote tumor progression, drug resistance, and metastasis. Although circulating tumor cells are studied as prognostic and diagnostic markers, little is known about other circulating cells and their association with PCa metastasis. Here, we explored the presence of circulating CAFs (cCAFs) in metastatic castration‐naïve prostate cancer (mCNPC) patients. cCAFs were stained with fibroblast activation protein (FAP), epithelial cell adhesion molecule (EpCAM), and receptor‐type tyrosine‐protein phosphatase C (CD45), then FAP^+^EpCAM^−^ cCAFs were enumerated and sorted using fluorescence‐activated cell sorting. FAP^+^EpCAM^−^ cCAFs ranged from 60 to 776 (389 mean ± 229 SD) per 2 × 10^8^ mononuclear cells, whereas, in healthy donors, FAP^+^ EpCAM^−^ cCAFs ranged from 0 to 71 (28 mean ± 22 SD). The mCNPC‐derived cCAFs showed positivity for vimentin and intracellular collagen‐I. They were viable and functional after sorting, as confirmed by single‐cell collagen‐I secretion after 48 h of culturing. Two cCAF subpopulations, FAP^+^CD45^−^ and FAP^+^CD45^+^, were identified, both expressing collagen‐I and vimentin, but with distinctly different morphologies. Collectively, this study demonstrates the presence of functional and viable circulating CAFs in mCNPC patients, suggesting the role of these cells in prostate cancer.

## Graphical abstract

Cancer‐associated fibroblasts (CAFs) promote cancer growth, invasion (metastasis), and drug resistance. Here, we identified functional and diverse circulating CAFs (cCAFs) in patients with metastatic prostate cancer (mPCa). cCAFs were found in higher numbers and were functional and diverse in mPCa patients versus healthy individuals, suggesting their role in cancer progression. Understanding cCAFs could lead to new treatment options for mPCa.

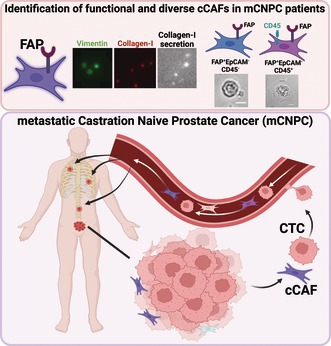

**Abbreviations**
AF: Alexa Fluor
apCAF: antigen‐presenting Cancer‐Associated Fibroblasts
BSA: bovine serum albumin
CAFs: cancer‐associated fibroblasts
Cav1: caveolin 1
cCAFs: circulating Cancer‐Associated Fibroblasts
CCL2: CC chemokine Ligand 2
CRPC: castration resistant prostate cancer
CTCs: circulating tumor cells
DLA: diagnostic leukapheresis
ECM: extracellular matrix
EMT: epithelial to mesenchymal transition
EpCAM: epithelial cell adhesion molecule
FACS: fluorescent activated cell sorting
FAP: fibroblast‐activated protein
FBS: Fetal Bovine Serum
FN1: FibroNectin 1
HPrFs: human prostate fibroblasts
iCAF: immune‐like cancer‐associated fibroblasts
iPSA: initial prostate specific antigen at primary diagnosis
ITGA5: integrin subunit alpha 5
LNCaP: lymph node carcinoma of the prostate
mCNPC: metastatic Castration‐naïve prostate cancer
MNCs: mononuclear cells
myCAF: myofibroblast‐like cancer‐associated fibroblasts
PBMCs: peripheral blood mononuclear cells
PBS: phosphate buffered saline
PCa: prostate cancer
PET: positron emission tomography
PSMA: prostate specific membrane antigen
PVDF: polyvinylidene difluoride
RNA: RiboNucleic Acid
TGFβ: transforming growth factor beta
TME: tumor microenvironment
α‐SMA: alpha‐smooth muscle actin

## 1 Introduction

Prostate cancer (PCa) is a common diagnosed malignancy in men, with over 1 million new cases diagnosed worldwide each year [1, 2]. Over the past 20 years, great advances were made in early diagnosis and novel treatment regimens, leading to a decreasing mortality rate. Despite these improvements, PCa remains the most dominant male malignancy worldwide. PCa is a highly metastatic tumor, and metastatic prostate cancer (mPCa) accounts for estimated 400 000 deaths annually [3]. While the 5‐year survival rate of localized PCa is almost 100%, for mPCa it is only 30% [4]. Additionally, patients with metastasis at initial diagnosis often have a more aggressive tumor and shorter overall survival compared to patients diagnosed with metastasis years after the initial primary tumor detection [5]. Therefore, early identification of patients at risk for developing lethal metastatic tumors, as well as better curative treatment options for these patients are necessary.

Solid tumors including PCa heavily depend on their tumor microenvironment (TME) for tumor growth and progression to a metastatic stage. Within this TME, cancer‐associated fibroblasts (CAFs) are one of the most abundant cell types that influence the behavior of tumor cells and other stromal cells [6]. They promote tumor growth by stimulating tumor cell proliferation and inhibiting apoptotic signals and anti‐tumor immune responses [7]. Moreover, they secrete extracellular matrix (ECM) proteins that favor tumor invasiveness and inhibit penetration of chemotherapeutics [8, 9].

In PCa, CAFs have also shown to play a major role in tumor metastasis and treatment response. Co‐culture of CAFs with tumorigenic PCa cells resulted in tumor cell growth, and eventually bone metastasis *in vivo* [10, 11]. Increasing evidence suggests that the specific crosstalk between CAFs and cancer cells determines the metastatic potential and metastatic location of a tumor. For example, in prostate adenocarcinoma, the tumor most frequently metastasizes to the bone, while in neuroendocrine prostate cancer primarily lung or liver metastases are observed [12]. This ‘seed and soil’ paradigm (proposed by Paget in 1889) suggests that the stroma of the metastatic site provides optimal microenvironment for the circulating PCa cells to survive and proliferate [13]. Moreover, androgen deprivation therapy – the standard treatment for metastatic PCa – leads to a phenotypical shift in CAFs, which contributes to ADT resistance in PCa cells [14].

Cancer‐associated fibroblasts are a heterogeneous population, varying in origin, phenotype, and functions. In recent years, several studies have investigated CAFs in PCa using single‐cell RNA seq [15, 16, 17, 18, 19, 20, 21, 22, 23, 24, 25]. In one study, three biologically distinct CAF subpopulations were identified in PCa with specific functions within the TME: myofibroblast‐like CAFs (myCAFs), immune and inflammatory CAFs (iCAFs), and antigen‐presenting CAFs (apCAFs) [15]. Using the gene set enrichment analysis (GSEA), they found that myCAFs are involved in epithelial to mesenchymal transition (EMT), angiogenesis and transforming growth factor beta (TGFβ) signaling; iCAFs in complement and coagulation cascades; and apCAFs in antigen processing and presentation [15]. iCAFs were also found to produce high amounts of chemotactic chemokines such as CC chemokine ligand 2 (CCL2), suggesting their involvement in the recruitment of immune cells particularly myeloid cells [26]. In another study, eight CAF subtypes were identified of which αSMA^+^CAV1^+^ CAFs‐C0 and FN1^+^FAP^+^ CAFs‐C1 were the two most abundant CAF subtypes with distinct molecular characteristics and biological functions in prostate cancer and bone metastasis. It was shown that CAFs‐C1 was associated with poor prognosis, castration resistance, and resistance to immune check point inhibitors [16]. Moreover, due to the distinctive features of CAFs, CAF gene expression profiles are used to develop tools for prognosing PCa biochemical recurrence [16, 27].

In mPCa, circulating tumor cells (CTCs) have been extensively studied as potential diagnostic and prognostic markers [28, 29, 30]. High CTC counts are associated with poor outcome, and monitoring their frequency serves as a treatment response marker [31, 32]. However, little is known about the other circulating cells in (m)PCa. Given the dependency of PCa cells on their TME, and the major role of CAFs in cancer metastasis, we hypothesized that – besides CTCs – CAFs can also be found in the circulation of PCa patients.

Therefore, in this study, we first developed a workflow that enables the detection of CAFs in circulation using a specific CAF marker, fibroblast‐activated protein (FAP) [33]. Then, we explored the presence of circulating CAFs (cCAFs) in diagnostic leukapheresis (DLA) aliquots of 2 × 10^8^ mononuclear cells (MNCs) from 18 metastatic castration‐naïve prostate cancer (mCNPC) patients and in the peripheral blood mononuclear cell (PBMC) fraction of 12 healthy controls, normalized to 2 × 10^8^ MNCs. We sorted the FAP^+^ cCAFs from mCNPC patients and evaluated their phenotype using collagen type I (collagen‐I) and vimentin immunofluorescent staining, as well as extracellular collagen‐I secretion. Finally, we identified two cCAF subpopulations, CD45‐positive and CD45‐negative, which were distinctly different in size and morphology.

## 2 Materials and methods

### 2.1 Cells

Human Prostate Fibroblasts (HPrFs), isolated from human prostate tissue, cryopreserved at P1, are purchased from ScienCell Research Laboratories (Catalog no. 4430, Carlsbad, CA, USA). They were cultured on poly‐l‐lysine (ScienCell) coated cell culture flasks (2 μg·cm^−2^) in fibroblast medium (Catalog no. 2301, ScienCell), supplemented with 1% Fibroblast Growth Supplement (FGS, ScienCell), 2% fetal bovine serum (FBS, ScienCell) and antibiotics (50 U·mL^−1^ Penicillin and 50 μg·mL^−1^ streptomycin, ScienCell). Human prostate cancer cell line, LNCaP (RRID:CVCL_0395), obtained from ATCC (Manassa, VA, USA), was cultured in RPMI1640 (Lonza, Verviers, Belgium) supplemented with 10% FBS (Lonza) and antibiotics (50 U·mL^−1^ Penicillin and 50 μg·mL^−1^ streptomycin, Lonza). Cells were cultured at 37 °C, 5% CO_2_ and trypsinized when reaching 70–80% confluency using 0.05% trypsin–EDTA (Gibco, Life Technologies, Waltham, MA, USA). HPrFs were cultured and used until passage P10 (low passage), while LNCaPs were cultured and used until P80 (high passage). All the cells were tested to exclude mycoplasma contamination using the Universal Mycoplasma Detection Kit (ATCC) thus ensuring that all the experiments were performed with mycoplasma‐free cells. Moreover, in our research, we adhere to strict laboratory practices to ensure the integrity and authenticity of our cell lines. Our standard protocol includes monitoring characteristics such as morphology, growth patterns, and specific marker expression, with epithelial adhesion molecule (EpCAM) expression (for the LNCaPs) serving as a key indicator in our study. We are confident about the reliability of our methods and the validity of the data produced from these cells used in this study.

### 2.2 Optimization of the workflows

To validate FAP surface expression on prostate fibroblasts, 2 × 10^5^ HPrFs were incubated with 5 μm CellTracker green dye (Invitrogen, Carlsbad, CA, USA) for 30 min at 37 °C. After washing through centrifugation, cells were resuspended in PBS with 2% FBS, spiked in 5 × 10^7^ MNCs, obtained from healthy volunteers, and stained with FAP‐AF647 (Table S1) for 1 h at 4 °C. Thereafter, the stained cells were washed again by centrifugation, and resuspended in PBS with 2% FBS. FITC and APC fluorescent signals of 50 000 cells were measured on the flow cytometer (FACS Aria II, BD Biosciences, Franklin Lakes, NJ, USA).

To optimize the cCAF isolation protocol, 2 × 10^5^ HPrFs and 2 × 10^5^ LNCaPs were spiked in 5 × 10^7^ MNCs freshly isolated from healthy volunteers. The cell suspension was stained with FAP‐AF647, epithelial adhesion molecule (EpCAM)‐PE and CD45‐AF488 (Table S1) for 1 h at 4 °C. After staining, cells were washed by centrifugation and resuspended in 2% FBS in PBS. Before enumeration through flow cytometry, aggregates were removed from the sample by straining it to a 70 μm cell strainer (Miltenyi BioTec Inc, San Fransisco, CA, USA). Flow cytometry parameters and compensation were adjusted to sort the cell populations of interest.

### 2.3 Patients and controls

Eighteen metastatic castration‐naïve prostate cancer (mCNPC) patients before starting with the androgen deprivation therapy (ADT) are included in this study. Biochemical markers analyses and prostate cancer staging was performed according to the standard of care in the Netherlands (in consultation with the treating physician). The patients participated in the PICTURES study, in which mCNPC patients with ≥ 3 CTC per 7.5 mL of blood as assessed by standard CellSearch analysis, underwent Diagnostic Leukapheresis (DLA) to harbor sufficient CTCs for an extensive CTC characterization. Leukapheresis was performed using a Terumo Spectra Optia according to the optimized procedure described by Mout et al. [34]. The study methodologies conformed to the standards set by the Declaration of Helsinki. All the study participants signed informed consent forms in accordance with the Helsinki declaration. The study and all the protocols were approved by the Medical Ethics Committee of the PICTURES study (MEC 2020‐0422, Erasmus MC). The written informed consent was obtained before any study procedures were performed. Healthy blood samples (40 mL, *n* = 12, of which three were age‐ and sex‐matched i.e., males aged above 50 years), were collected in standard EDTA tubes, from anonymized healthy volunteers at the University of Twente. From healthy donors' blood, the PBMC fraction was collected using Ficoll‐Plaque plus (1.077 g·mL^−1^) density gradient separation (VWR, Amsterdam, The Netherlands). In agreement with the Declaration of Helsinki, informed consent was obtained from all volunteers and the used blood collection procedure was approved by the local Medical Research Ethics Committee (MEC K11‐23, Twente). Diagnostic Leukapheresis (DLA) samples from mCNPC patients were collected at the Erasmus MC, while the blood samples from healthy donors were collected at the University of Twente. The samples were collected from January 2022 to December 2023, and processed‐analyzed as mentioned below.

### 2.4 CTC enumeration

CTCs were enumerated at the Erasmus MC in the DLA aliquots of 2 × 10^8^ MNCs from 18 mCNPC patients within 48 h after the DLA procedure was performed. DLA aliquots were processed with CellTracks Autoprep® using the CellSearch Circulating Tumor Cell kit and analyzed on the CellTracks Analyzer II® (Menarini, Huntingdon Valley, PA, USA) as previously described [35].

### 2.5 cCAFs enumeration

cCAFs were enumerated in the DLA aliquots of 2 × 10^8^ MNCs and transported overnight at room temperature to the MCBP laboratory at the University of Twente from the same 18 mCNPC patients in which the CTC enumeration was performed (see above). cCAFs were enumerated by flow cytometry, after immunofluorescent labeling with FAP‐AF647, EpCAM‐PE and CD45‐AF488 (Table S1) for 1 h at 4 °C. After staining, cells were washed by centrifugation and resuspended in 2% FBS in PBS. Before enumeration through flow cytometry, aggregates were removed from the sample by straining it through a 70 μm cell strainer (Miltenyi BioTec).

### 2.6 Fluorescence activated cell sorting

Each mCNPC sample (containing 2 × 10^8^ MNCs) prepared as mentioned above, was passed through the flow cytometer completely, after applying a threshold on forward scatter and FAP positivity. For the healthy donors, FAP^+^ counts were normalized to 2 × 10^8^ MNCs present in a DLA aliquot. In 10 patient samples, FAP positive cells were sorted into 2% FBS/PBS pre‐coated sterile FACS tubes for further downstream analysis. In one sample, FAP^+^ CD45^−^ and FAP^+^ CD45^+^ cells were sorted separately into two different 2% FBS/PBS pre‐coated sterile FACS tubes, for morphological analysis. In four healthy volunteers, FAP positive cells were sorted and pooled together for further analysis.

### 2.7 Microscopy

In four mCNPC samples and a pooled healthy volunteer sample, sorted cells were spun down (370 ***g***) on pre‐labeled microscope slides by Cytospin (Hettich, Tuttlingen, Germany), and fixed with 4% paraformaldehyde (Merck, Sigma, St. Louis, MO, USA) for 15 min at room temperature. After rinsing with PBS, cells were permeabilized with cold methanol (Sigma) for 10 min at −20 °C, washed twice with 0.05% Tween20 (Sigma) containing PBS, and blocked for 30 min in 5% bovine serum albumin (BSA, Sigma) containing PBS. Cells were stained with primary antibodies (goat anti‐human Collagen‐I and rabbit anti‐human Vimentin) overnight at 4 °C (1 : 100) (Table S2). Cells were then incubated with Alexa‐488‐ and Alexa‐594‐labeled secondary antibody (1 : 200) (Table S2). Thereafter, the cells were mounted using DAPI‐containing antifade mounting medium (Merck, Sigma). The staining was visualized, and images were captured using Nikon Eclipse T*i* inverted fluorescence microscope (Nikon, Minato, Tokyo, Japan).

### 2.8 Collagen‐I secretion

The method for the detection of single cell collagen‐I secretion was described earlier [36]. Briefly, low fluorescent polyvinylidene difluoride (PVDF) membranes (BioRad, Lunteren, The Netherlands) were coated with human collagen‐I coating antibody (4 μg·mL^−1^) from the human collagen‐I duoset ELISA kit (R&D Systems, Abingdon, UK), overnight at 4 °C. Membranes were blocked with sterile 3% BSA containing PBS for 1.5 h, after which culture medium was added on top of the membranes. Sorted FAP positive cells were cultured – with or without 10 ng·mL^−1^ transforming growth factor β (TGFβ, R&D Systems) – on top of the collagen‐I coated PVDF membranes, in fibroblasts medium supplemented with 1% FGS, 2% FBS and antibiotics, for 2 days at 37 °C, 5% CO_2_. Cells were washed off the membranes by incubation with 0.05% Tween‐20 containing PBS for 20 min on a plate shaker. Thereafter, the membranes were washed in 1% BSA containing PBS and incubated with the biotinylated collagen‐I primary detection antibody (100 ng·mL^−1^) from the corresponding DuoSet ELISA kit for 2 h on a plate shaker. After washing, the membranes were incubated with streptavidin‐PE for 1 h in the dark, washed with MilliQ water, dried and imaged using scanning software on the Nikon fluorescent microscope. Montages of the images were created using imagej software (National Institute of Health, Bethesda, MD, USA), and secretion spots per membrane were counted manually. The percentage of cells secreting collagen‐I was calculated as the number of spots per membrane divided by the number of cells/events plated. The number of cells/events plated is retrieved from the total events sorted by the FACS sorter.

### 2.9 Statistics

Graphs and statistical analysis were performed using graphpad prism version 10.0.2 (GraphPad Prism Software, La Jolla, CA, USA). Cell counts are presented as mean ± standard deviation (SD). Unpaired Student's *t*‐test with correction for skewed gaussian distribution (Mann–Whitney) was used to compare FAP^+^ counts (cCAFs) of mCNPC patients and healthy donors. Comparison between control and TGFβ stimulation was analyzed using unpaired Student's *t*‐test and the correlation between CTCs, and cCAFs was analyzed using two‐tailed Spearman rho correlation analysis using graphpad prism software. Differences were considered statistically significant when *P < 0.05, *P < 0.01 and ****P < 0.0001. All experiments were performed at least three times independently.

## 3 Results

### 3.1 Establishing a workflow for cCAF isolation

**Fig. 1:**
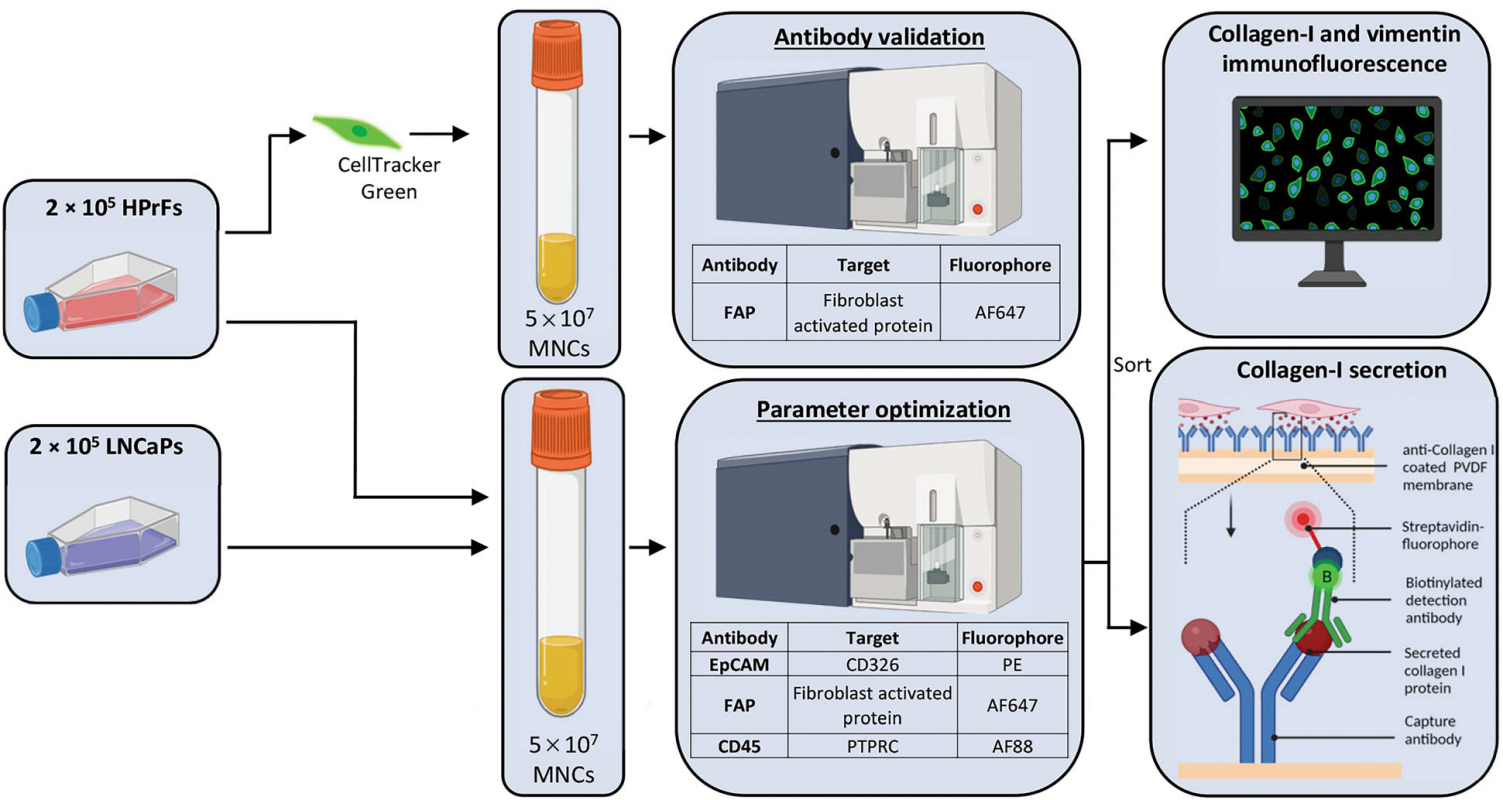
Schematic showing the workflow, using human cell lines, for the isolation and (functional) characterization of circulating cancer‐associated fibroblasts. 2 × 10^5^ HPrFs (representing circulating cancer‐associated fibroblasts, cCAFs) and 2 × 10^5^ LNCaPs (representing circulating tumor cells, CTCs) were spiked into 5 × 10^7^ freshly isolated human MNCs. To discriminate between CTCs and cCAFs and to isolate the cCAFs from the MNC fraction, the samples were stained with a combination of the antibodies targeting cell‐specific surface markers [FAP (for cCAFs), CD45 (PTPRC, for MNCs) and Epithelial Cell adhesion molecule (EpCAM, for CTCs)]. After labeling, the complete sample of 5 × 10^7^ MNCs ran through the FACS, and FAP^+/−^ cells were sorted for further analysis, including intracellular immunofluorescent staining for vimentin (a mesenchymal cell marker) and collagen‐I (an ECM marker), and extracellular collagen‐I secretion. AF, Alexa Fluor; CD, cluster of differentiation; EpCAM, epithelial cell adhesion molecule; FAP, fibroblast‐activated protein; HPrFs, human prostate fibroblasts; LNCaPs, lymph node carcinoma of the prostate; MNCs, mono nuclear cells; PE, Phycoerythrin; PTPRC, protein tyrosine phosphatase receptor type C; PVDF, Polyvinylidene difluoride.

For the isolation of cCAFs, we created a workflow depicted in Fig. 1 consisting of immunofluorescence labeling with subsequent flow‐activated cell sorting and characterization of the sorted cell populations. For the identification of CAFs, a specific CAF cell surface marker is essential. Fibroblast‐activated protein (FAP) is a specific cell surface receptor associated with activated CAFs and is involved in stromal‐epithelial interactions. Moreover, recent studies revealed increased FAP expression in CAFs of PCa that correlate with PCa prognosis [16, 37, 38], and FAP targeting ligands are being evaluated as therapeutic carriers in multiple cancer patient trials [39, 40]. The presence of FAP surface receptor on CellTracker‐labeled human prostate fibroblasts (HPrFs) spiked in 5 × 10^7^ human mononuclear cells (MNCs) was evaluated using flow cytometry which showed FAP positivity in 95.6 ± 1.4% of CellTracker‐labeled HPrFs suggesting that the majority/all of HPrFs express FAP (Fig. S1).

Next, to mimic a patient sample, 2 × 10^5^ HPrFs (representing circulating CAFs, cCAFs) and 2 × 10^5^ LNCaPs (representing circulating tumor cells, CTCs) were spiked into 5 × 10^7^ freshly isolated human MNCs (Fig. 1). To discriminate between CTCs and cCAFs and to isolate the cCAFs from the MNC fraction, the samples were immunofluorescently labeled with a combination of the antibodies targeting cell‐specific surface markers [FAP (for cCAFs), CD45 (for leukocytes) and Epithelial Cell adhesion molecule (EpCAM, for CTCs)]. After labeling, the complete sample of 5 × 10^7^ MNCs was ran through the FACS, and FAP^+/−^ cells were sorted for further analysis, including intracellular immunofluorescent staining for vimentin (a mesenchymal cell marker) and collagen‐I (an ECM marker), and extracellular collagen‐I secretion (Fig. 1).

**Fig. 2:**
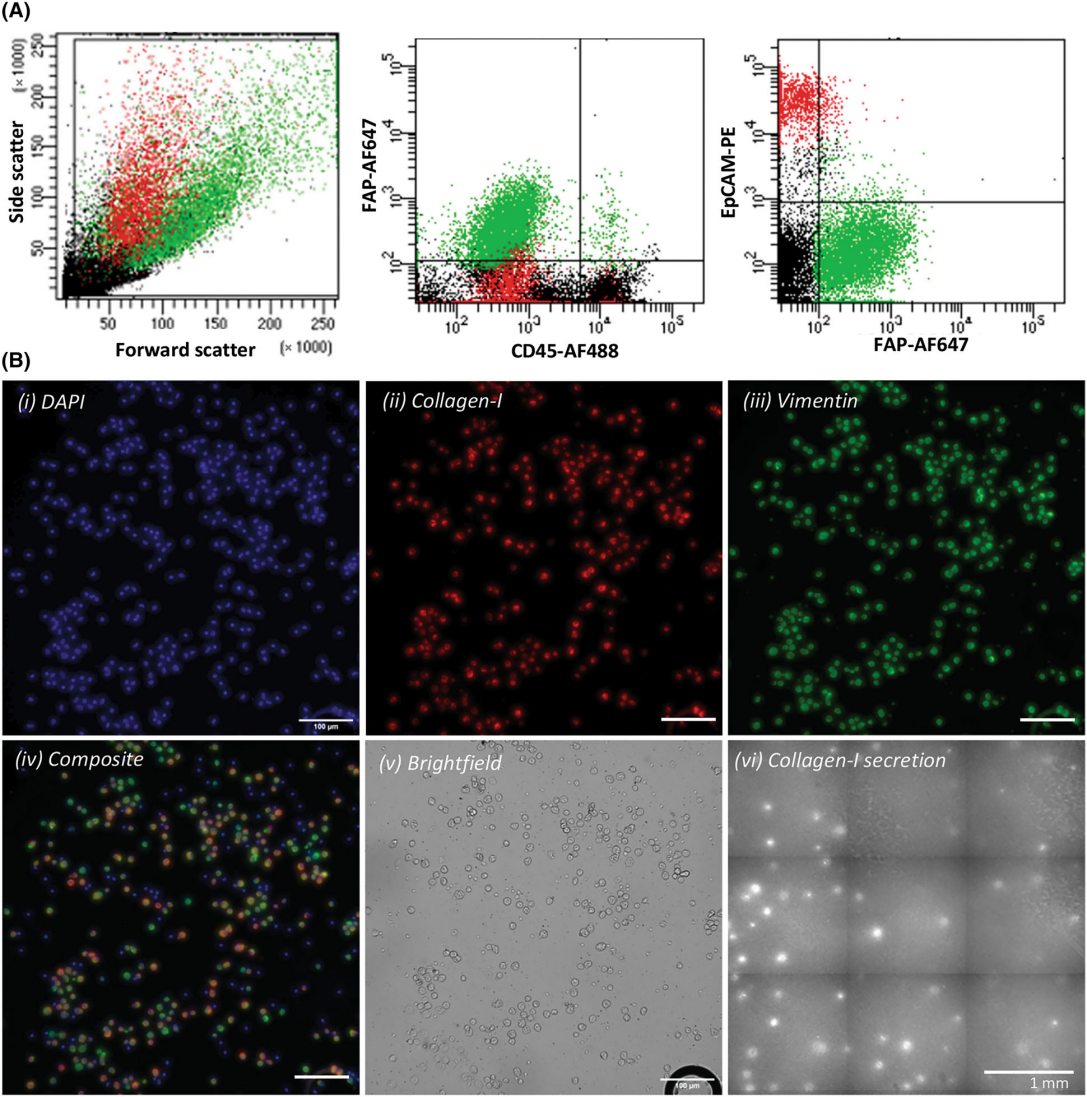
Identification and (functional) characterization of HPrFs that were spiked in MNCs of healthy volunteers and were isolated using the workflow depicted in Fig. 1. (A) Representative flow plots of HPrFs (green) and LNCaPs (red) spiked in the MNCs of healthy volunteers, stained with FAP‐AF647, CD45‐AF488 and EpCAM‐PE. FAP‐negative events/cells are shown in black. (B) Representative images (4×, Scale bar = 100 μm, *n* = 3) of sorted HPrFs showing (i) DAPI‐stained nuclei (blue), (ii) intracellular collagen‐I staining (red), (iii) intracellular vimentin staining (green) and (iv) a composite image; (v) cell morphology and density (brightfield), and (vi) collagen‐I secretion captured on the PVDF membrane (scale bar = 1 mm). AF, Alexa Fluor; CD, cluster of differentiation; EpCAM, epithelial cell adhesion molecule; FAP, fibroblast‐activated protein; HPrFs, human prostate fibroblasts; LNCaPs, lymph node carcinoma of the prostate; MNCs, mono nuclear cells; PE, Phycoerythrin.

After immunofluorescent labeling, the FAP^+^ EpCAM^−^ HPrFs (Fig. 2A), and FAP^−^ EpCAM^+^ LNCaPs (Fig. S2A) were sorted. Immunofluorescent staining of the sorted cells revealed that the FAP^+^ EpCAM^−^ HPrFs showed positive intracellular staining of collagen‐I and vimentin (Fig. 2B), while the FAP^−^ EpCAM^+^ LNCaPs showed negative staining for both markers (Fig. S2B).

Furthermore, to evaluate the functionality of sorted fibroblasts, collagen‐I secretion of sorted FAP^+^ EpCAM^−^ HPrFs and sorted FAP^−^ EpCAM^+^ LNCaPs was measured after 2 days of culture. This was done by culturing the sorted cells on the PVDF membranes pre‐coated with the collagen‐I antibody.

After removal of the cells, secreted collagen‐I captured on the membrane was visualized using a biotin/streptavidin antibody combination, as described previously [36], where each spot on the membrane corresponds to the collagen‐I protein secreted by a single cell. In Fig. 2B, the abundance of collagen‐I spots indicates that the FAP^+^ EpCAM^−^ HPrFs secrete collagen‐I, while the FAP^−^ EpCAM^+^ LNCaPs do not secrete collagen‐I (Fig. S2B).

### 3.2 Identification of circulating cancer‐associated fibroblasts (cCAFs) in metastatic castration‐naïve prostate cancer (mCNPC) patients

**Fig. 3:**
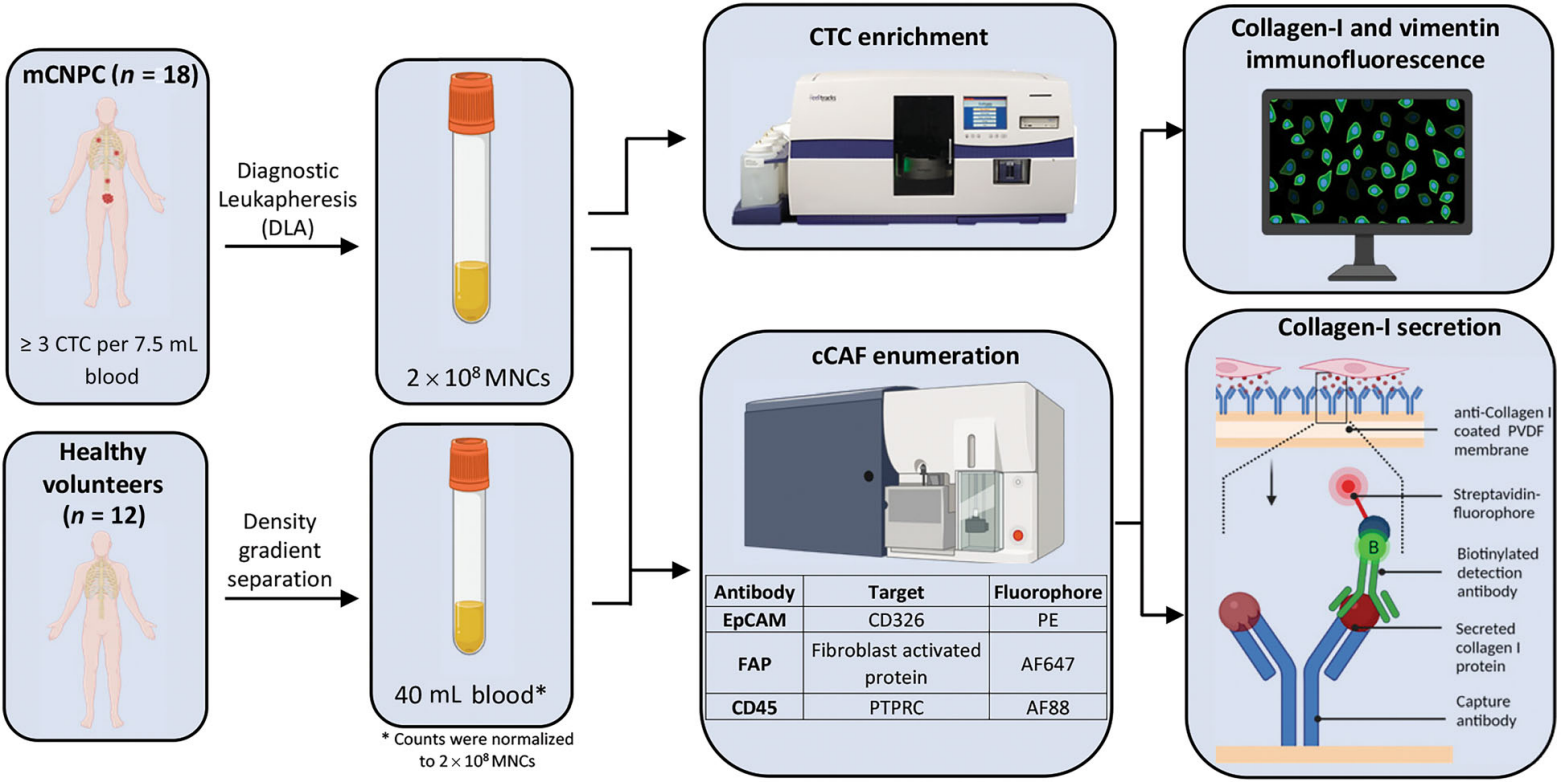
Schematic showing the workflow for the isolation (and functional characterization) of circulating tumor cells (CTCs) and circulating cancer‐associated fibroblasts (cCAFs) from metastatic castration‐naïve prostate cancer (mCNPC) patients and healthy controls. Diagnostic LeukApheresis (DLA) samples from 18 mCNPC patients were subjected to CTC enrichment using a CellSearch system. Moreover, DLA from 18 mCNPC patients (aliquots of 2 × 10^8^ MNCs) and PBMCs from 12 healthy donors (40 mL blood, normalized to 2 × 10^8^ MNCs), were immunofluorescently labeled with EpCAM‐PE, FAP‐AF647, and CD45‐AF488 and analyzed by flow cytometry using a threshold on forward scatter and FAP‐AF647. Sample acquisition was halted when the complete sample passed the flow cytometer. FAP positive cells were sorted and subjected to intracellular staining of collagen‐I and vimentin, and extracellular collagen‐I secretion analysis. AF, Alexa Fluor; cCAF, circulating cancer‐associated fibroblast; CD, cluster of differentiation; CTC, circulating tumor cell; EpCAM, epithelial cell adhesion molecule; FAP, fibroblast activated protein; mCNPC, metastatic Castration‐Naïve Prostate Cancer; MNCs, mono nuclear cells; PE, Phycoerythrin; PTPRC, Protein Tyrosine Phosphatase receptor type C; PVDF, Polyvinylidene difluoride.

Diagnostic leukapheresis (DLA), a process that enriches the mononuclear cells by continuous centrifugation of blood [34], was performed on metastatic castration‐naïve prostate cancer (mCNPC) patients with ≥ 3 CTCs per 7.5 mL of blood. The baseline characteristics of the CNPC patients included in this study are provided in Table 1. DLA from 18 mCNPC patients were subjected to CTC enrichment using a CellSearch system. Moreover, DLA from 18 mCNPC patients (aliquots of 2 × 10^8^ MNCs) as well as PBMCs from 12 healthy donors (40 mL blood, normalized to 2 × 10^8^ MNCs), were immunofluorescently labeled with EpCAM‐PE, FAP‐AF647, and CD45‐AF488 and analyzed by flow cytometry using a threshold on forward scatter and FAP‐AF647. Sample acquisition was halted when the complete sample passed the flow cytometer. FAP positive cells were sorted and subjected to intracellular staining of collagen‐I and vimentin, and extracellular collagen‐I secretion analysis as illustrated in Fig. 3.

**Table 1:** Baseline patient characteristics. M1a, lymph node metastasis; M1b, bone metastasis; M1c, visceral metastasis (with or without bone metastasis); Mo, no metastasis; PSA, Prostate‐Specific Antigen; Range, minimum – maximum; SD, Standard Deviation; WHO PS, World Health Organization Performance Score.

	CNPC (*n* = 18)
Age at registration	
Median (range) – years	70 (58–78)
WHO PS at registration – no. (%)	
−0	12 (66.7%)
−1	6 (33.3%)
Initial PSA at primary diagnosis – μg/L	
Mean ± SD	508.3 ± 1088.6
Median (range)	128.25 (14.9–4682)
Hemoglobin in g/L	
Mean ± SD	8.0 ± 1.3
Median (range)	7.9 (5.7–9.9)
Alkaline phosphatase – IU/L	
Mean ± SD	452.7 ± 592.8
Median (range)	316.5 (83–2431)
Lactate dehydrogenase – IU/L	
Mean ± SD	293.4 ± 124.7
Median (range)	227 (199–644)
Albumin – g/L	
Mean ± SD	39.2 ± 6.5
Median (range)	40 (28–47)
Gleason score by diagnosis	
Median (range)	8 (6–10)
M‐stage at diagnosis	
M0	3 (16.7%)
M1a, M1b, M1c	15 (83.3%)

**Fig. 4:**
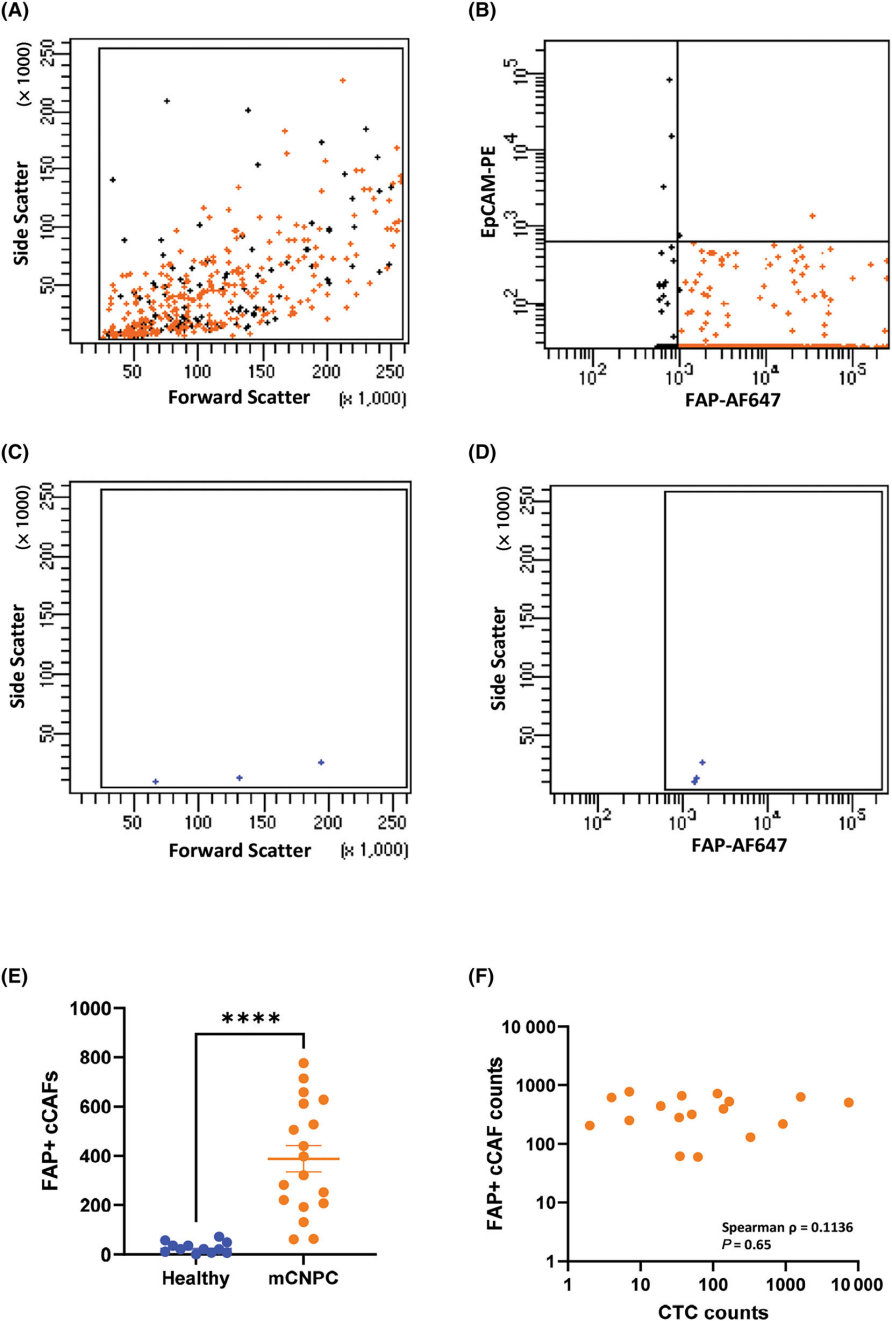
Circulating Cancer‐associated fibroblasts (cCAFs) found in mCNPC patients versus healthy volunteers. (A, B) Representative scatter plot showing (A) forward versus side scatter and (B) FAP‐AF647 versus EpCAM‐PE staining of FAP positive cells in a mCNPC patient, *n* = 18. FAP‐negative events/cells are shown in black. (C, D) Representative scatter plot showing (C) forward versus side scatter and (D) FAP‐AF647 versus side scatter staining of FAP positive cells in a healthy donor, *n* = 12. (E) Bar graph showing cCAF counts of healthy donors and mCNPC patients (normalized to 2 × 10^8^ mononuclear cells); error bars indicate standard error of mean (SEM); statistical analysis was performed by Mann–Whitney test; ****P < 0.0001. (F) Spearman correlation plot between cCAF counts (based on flow cytometry) versus CTC counts (based on the CellSearch system) normalized to 2 × 10^8^ mononuclear cells. Spearman ρ denotes Spearman correlation and *P* value denotes the statistical significance; AF, Alexa Fluor; cCAF, circulating Cancer‐associated fibroblast; CTC, circulating tumor cell; EpCAM, epithelial cell adhesion molecule; FAP, fibroblast activated protein; mCNPC, metastatic Castration‐naïve Prostate Cancer; PE, Phycoerythrin.

In Fig. 4A–D, a typical flow cytometric analysis of a mCNPC patient sample (Fig. 4A,B) and a healthy volunteer sample (Fig. 4C,D) is shown. In the mCNPC patients, the flow cytometry analysis showed that the FAP‐positive population were EpCAM negative (Fig. 4B). In the healthy donors, a few FAP positive (EpCAM negative) events were observed, and no difference between age‐ and sex‐matched and the other healthy donors was evidenced. Moreover, the events that were FAP positive, were relatively small in the side scatter (Fig. 4C) and dim in the fluorescent intensity (Fig. 4D). In 18 mCNPC patients, cCAFs, defined as FAP^+^, ranged from 60 to 776 (mean 389 ± 229 SD) (Fig. 4E, Table S3), while in 12 healthy donors, FAP^+^ cells ranged from 0 to 71 (mean 28 ± 22 SD) (Fig. 4E, Table S3). Moreover, CTCs in the mCNPC patients ranged from 0 to 7463 (610 mean ± 1753 SD) (Table S3), and no significant correlation between CTCs and FAP^+^ cCAFs was observed (Fig. 4F). The total FAP^+^ cCAF and EpCAM^+^ CTC counts of these patients are included in Table 2.

**Table 2:** CTC and cCAF counts in CNPC patients. Range, minimum – maximum; SD, standard deviation.

	CNPC (*n* = 18)
CTC count^a^	
Mean ± SD	610 ± 1753
Median (range)	44 (0–7436)
Total FAP^+^ cCAF count^a^	
Mean ± SD	389 ± 229
Median (range)	360 (60–776)
FAP^+^ CD45^−^ cCAF count^a^	
Mean ± SD	192 ± 109
Median (range)	205 (19–382)
FAP^+^ CD45^+^ cCAF count^a^	
Mean ± SD	197 ± 152
Median (range)	117 (26–500)

a Normalized to 2 × 10^8^ mononuclear cells.

### 3.3 (Functional) characterization of circulating FAP ^+^ cancer‐associated fibroblasts

**Fig. 5:**
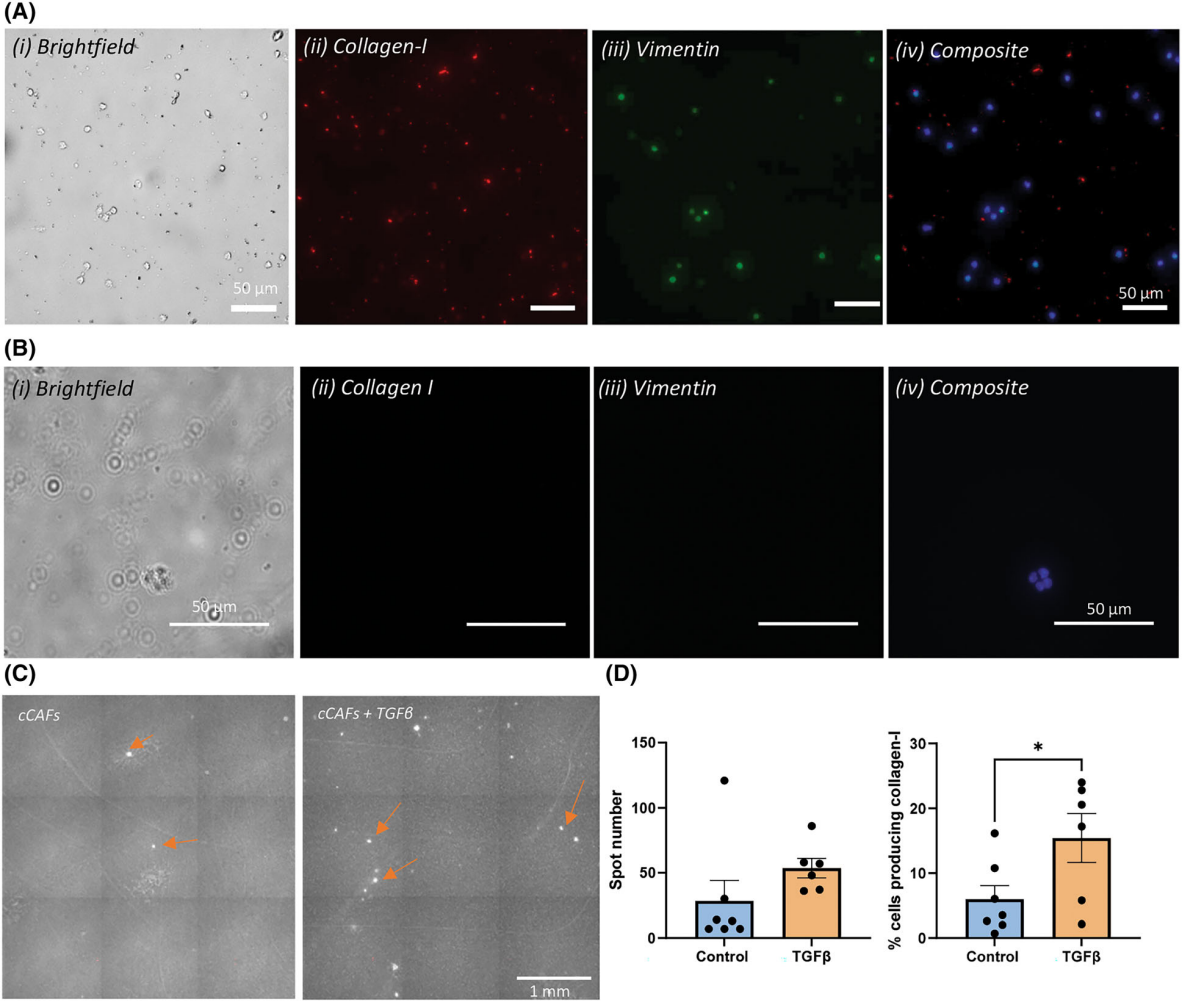
Characterization of circulating FAP^+^ cancer‐associated fibroblasts. (A, B) Representative images (10×, Scale bar = 50 μm, *n* = 4) showing (i) cell morphology and density (brightfield), (ii) intracellular collagen‐I staining (red), (iii) intracellular vimentin staining (green) and (iv) a composite image with DAPI‐stained nuclei (blue), of FAP^+^ cCAFs from (A) mCNPC patients and (B) healthy volunteers. (C) Collagen‐I secretion captured on the PVDF membrane (Scale bar = 1 mm). Arrows indicate single‐cell collagen‐I secretion. (D) Quantification from (C), *n* = 7. Error bars indicate standard error of mean (SEM); statistical analysis was performed using Students *t*‐test, *P < 0.05. cCAF, circulating Cancer‐associated fibroblast; TGFβ, Transforming Growth Factor beta.

FAP^+^ EpCAM^−^ events were sorted and cytospins of the sorted cells were immunofluorescently stained with anti‐collagen‐I and anti‐vimentin antibodies. The cCAFs derived from mCNPC patients appeared round and relatively smaller compared to HPrFs. 94 ± 5.9% of the cells expressed collagen‐I, and 67.7 ± 14.2% expressed both collagen‐I and vimentin (Fig. 5A). Furthermore, events sorted from healthy donors (*n* = 4) were pooled and stained for collagen‐I and vimentin. Here, we observed that the sorted cells from healthy donors were negative for vimentin and collagen‐I (Fig. 5B).

A main function of CAFs is the remodeling of the ECM through the secretion of various ECM proteins importantly collagens [41]. Here, activation of the TGFβ signaling pathway is of great importance, as it leads to the activation of CAFs, as well as EMT transition in cancer cells [42]. We therefore evaluated whether or not the sorted circulating CAFs were still capable of producing ECM components, such as collagen‐I, and if the cCAFs show responsiveness hence activation upon TGFβ treatment.

In seven of the 18 mCNPC samples, we cultured the sorted FAP^+^ cells on collagen‐I antibody pre‐coated membranes for 2 days, with or without TGFβ. Each spot on these membranes represents the collagen‐I protein secreted by a single cell (Fig. 5C). Without stimulus, cCAFs showed an average of 28 ± 41 spots per membrane. However, when these cells were stimulated with TGFβ, more spots i.e., an average of 54 ± 18 spots were observed per membrane indicating that cCAFs respond to TGFβ and the percentage of cells secreting collagen‐I significantly increases upon TGFβ stimulation (Fig. 5D).

### 3.4 Subpopulations of circulating fibroblasts

**Fig. 6:**
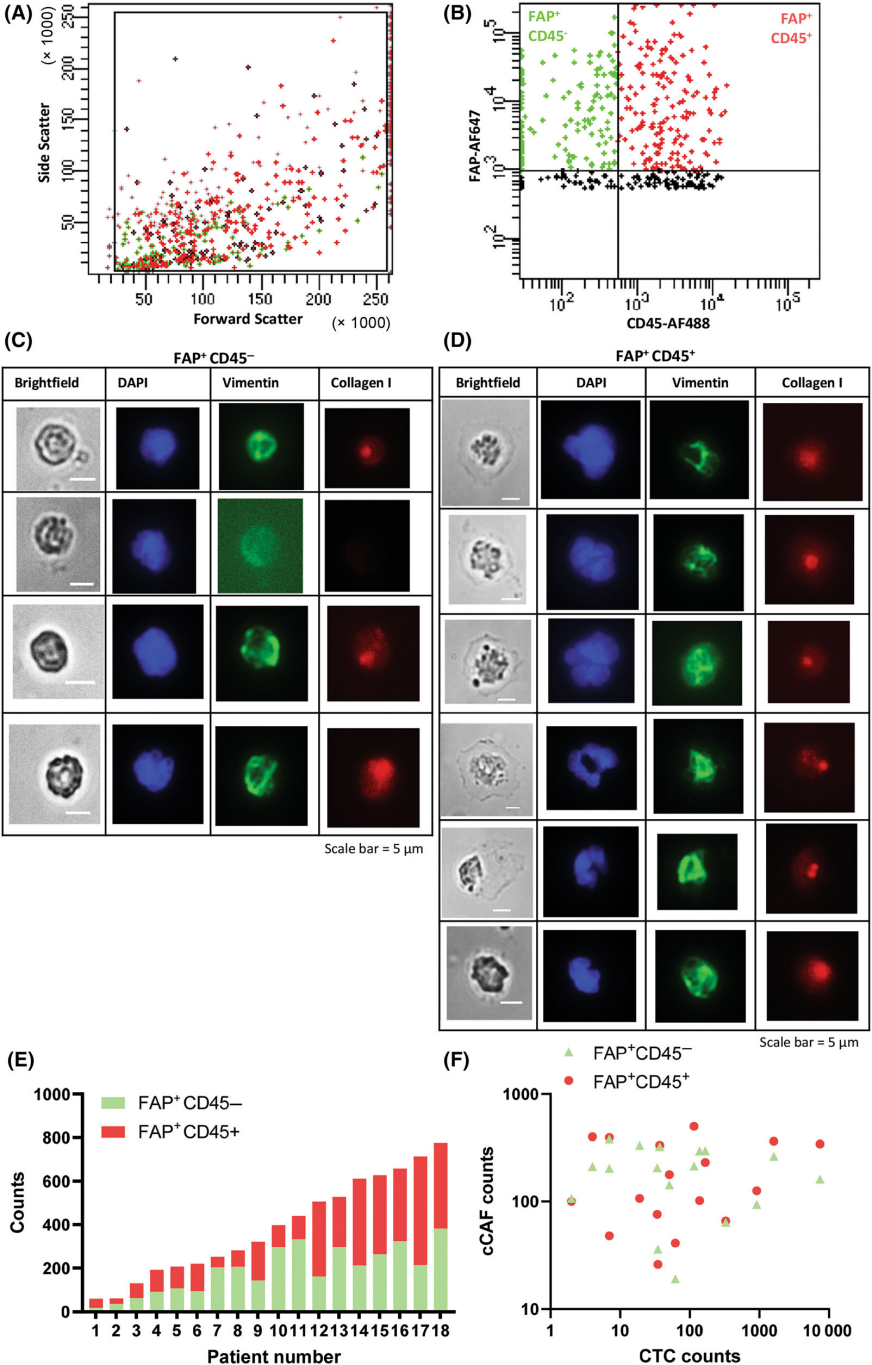
Identification of circulating cancer‐associated fibroblast subpopulations in mCNPC patients. (A, B) Scatterplots of forward versus side scatter (A) and CD45‐AF488 versus FAP‐AF647 (B), indicating a FAP^+^CD45^−^ (green) and a FAP^+^CD45^+^ (red) subpopulation. FAP‐negative events/cells are shown in black. (C, D) Brightfield, DAPI (blue), vimentin (green) and collagen‐I (red) stained images of sorted (C) FAP^+^CD45^−^ and (D) FAP^+^CD45^+^ cells, scale bar = 5 μm. (E) FAP^+^CD45^−^ and FAP^+^CD45^−^ counts (normalized to 2 × 10^8^ mononuclear cells) in 18 mCNPC patients and (F) Correlation between FAP^+^ subpopulations and the CTC counts (normalized to 2 × 10^8^ mononuclear cells) found in mCNPC patients (*n* = 18). AF, Alexa Fluor; cCAF, circulating Cancer‐associated fibroblast; CD45, cluster of differentiation 45; CTC, circulating tumor cell; DAPI, nuclear stain; FAP, fibroblast activated protein.

Further evaluation of the FACS data from mCNPC patients revealed two FAP positive subpopulations: one expressing CD45 (FAP^+^ CD45^+^) and the other not (FAP^+^ CD45^−^). A typical FACS plot is shown in Fig. 6A,B, in which the FAP^+^ CD45^−^ depicted in green, and the FAP^+^ CD45^+^ depicted in red (Fig. 6B). The forward and side scatter of the FAP^+^ CD45^−^ population were relatively small compared to the FAP^+^ CD45^+^ suggesting a difference in the morphology of these two subpopulations (Fig. 6A). To evaluate the differences between these two subpopulations, the two populations were sorted separately and their morphology, and intracellular collagen‐I and vimentin staining were evaluated (Fig. 6C,D). Both subpopulations expressed vimentin and collagen‐I. However, consistent with the flow cytometry data, the FAP^+^ CD45^−^ cCAFs were smaller in size, and had a round and compact morphology (Fig. 6C). On the other hand, the FAP^+^ CD45^+^ cCAFs were bigger, and the cytoplasm was spread out (Fig. 6D). The FAP^+^ CD45^−^ cCAFs ranged from 19 to 382 (192 mean ± 109 SD), and the FAP^+^ CD45^+^ cCAFs ranged from 26 to 500 (197 mean ± 152 SD). In healthy individuals, FAP^+^ CD45^−^ cCAFs ranged from 0 to 12 (5 mean ± 4 SD), and the FAP^+^ CD45^+^ cCAFs ranged from 0 to 65 (23 mean ± 21 SD). An overview of the FAP^+^ CD45^+^ cCAF and FAP^+^ CD45^−^ cCAF counts in mCNPC patients is given in Table 2, depicted in Fig. 6E, and provided per patient and healthy individual in Table S3. When comparing CTCs with cCAFs, no significant correlation between total FAP^+^ cCAF or cCAF (FAP^+^ CD45^+^ cCAF and FAP^+^ CD45^−^) subpopulations, and total CTCs in mCNPC patients could be observed (Fig. 6F). Moreover, no significant correlation between baseline patient characteristics and total FAP^+^ cCAF or cCAF subpopulations (FAP^+^ CD45^+^ cCAF and FAP^+^ CD45^−^) was observed. Lack of correlations could be attributed to the limited number of patients included in this study.

## 4 Discussion

In this paper, we show, for the first time, the presence of viable and functional FAP positive circulating CAFs in metastatic CNPC patients. CAFs are the major drivers in PCa that promote tumor growth, metastasis and resistance to therapy [6, 8]. Multiple studies indicated that CAFs form a supportive microenvironment at the pre‐metastatic niche where CAFs regulate ECM remodeling, activate tumor cells and other stromal cells, and create an immunosuppressive environment [43, 44].

Previous studies have shown the presence of circulating fibroblast‐like cells in other metastatic cancers. These studies report different markers to identify and characterize CAFs. Most commonly used CAF markers include FAP, α‐SMA, S100A4, platelet‐derived growth factor receptors (PDGFRα/β) or vimentin [44], while more markers are currently been identified e.g., ITGA5 [45]. For example, Ao et al. [46] reported FAP and α‐SMA positive cCAFs in the blood of metastatic breast cancer patients. Additionally, Lu et al. identified cCAFs in the blood of orthotopic breast tumor‐bearing mice, using FAP and ITGA5 as cCAF markers [45]. Pan et al. [16], used αSMA, CAV1, FN1, FAP, to identify two subtypes of CAFs. In this study, we used FAP to identify and isolate cCAFs as FAP overexpression correlates with higher risk of tumor invasion, lymph node metastasis, decreased overall survival and resistance to therapies [16, 47]. It is important to note that none of these markers are specific to CAFs and therefore further characterization of the CAFs are necessary to confirm CAF phenotype. We used vimentin (and intracellular and extracellular collagen) to confirm cCAFs phenotype which is shown to be expressed among the different CAF subsets in prostate cancer [15]. Jones et al. [48] also used vimentin to identify fibroblast‐like cells in the circulation of metastatic PCa patients by adding vimentin as an additional marker in the CellSearch system thereby identifying vimentin^+^ EpCAM^+^ cells. Vimentin is a mesenchymal cell marker and is not a fibroblast specific marker, as it is also present on cancer cells that have undergone EMT [49, 50]. Hence, most likely, they [48] identified CTCs that underwent EMT, clusters of CTCs with fibroblast‐like cells, or both. Previous study reported the rare clusters containing CTCs and FAP positive cCAFs in metastatic breast cancer patients that correlated with increased metastatic potential [51]. Very recently, Ortiz‐Otero et al. isolated and evaluated CTCs and cCAFs from patients with localized PCa [52]. Here, CTCs were defined as Cytokeratin^+^ CD45^−^, and cCAFs were defined as CD45^−^ α‐SMA^+^ positive. The CTC numbers reported in this study are however, extremely high (> 35 CTCs per ml of blood), making their CTC definition questionable. Moreover, cCAFs were enriched using a commercially available extraction kit with a general mesenchymal stem cell marker, making the cCAF enrichment not specific to fibroblast.

Single cell RNA sequencing revealed three heterogeneous CAF subpopulations in PCa, myCAFs (myofibroblast‐like CAFs; expressing high levels of activated fibroblast and ECM‐associated genes), iCAFs (immune‐like CAFS; expressing high levels of inflammatory markers) and apCAFs (antigen‐presenting CAFs; expressing antigen‐presenting genes) [15, 26]. A trajectory analysis revealed that the iCAFs might be an initial subpopulation of the CAFs [15]. Another study identified eight heterogenous CAF subtypes of which two subtypes (αSMA^+^ CAV1^+^ CAFs‐C0 and FN1^+^ FAP^+^ CAFs‐C1) (possibly myCAF subtypes) were more prevalent in PCa. Trajectory analysis indicated αSMA^+^ CAV1^+^ CAFs‐C0 as the initial population [16]. In our study, after isolation of cCAFs, positive vimentin staining confirmed the mesenchymal origin of our FAP positive cells. Moreover, we showed that these cells produce the ECM protein collagen‐I, and that they showed responsiveness to TGFβ. We therefore speculate that the cCAFs found in our mPCa cohort are myCAFs. This can be supported by Liang et al. [53], as they found a significant increase of myCAFs at the metastatic site, compared to the primary tumor. Moreover, at the bone metastatic site, collagen‐I is the most abundant structural ECM protein, and the pre‐metastatic niche is created out of a collagen matrix, with designated gaps for homing tumor cells [54]. PCa cells were found to attach better and undergo EMT when seeded on collagen‐I, indicating that a collagen‐I‐rich matrix might contribute to increased tumor aggressiveness [55]. Moreover, Pan et al. [16] suggested the FAP^+^ CAF subpopulation correlates with castration resistance and poor prognosis. Therefore, we speculate that the presence of these FAP^+^ cCAFs in the blood contributes to creating a tumor‐friendly microenvironment at the metastatic site. Thus, further molecular and functional analysis of FAP^+^ cCAFs is imperative to understand the role of these cells in prostate and other cancer types, and in metastasis. Since the number of cCAFs isolated from mCNPC patients is relatively low, performing different analyses can be challenging. Most of the functional assays therefore should be performed at the single‐cell level. For example, single‐cell RNA sequencing and single‐cell secretome analysis will further delineate the heterogeneity in cCAFs and can correlate the gene/protein signatures with the specific functions in circulation [56]. Immunostainings using other CAF‐associated markers (αSMA, CAV1, FN1, PDGFR, FSP1, ITGA5) could be performed to further examine the heterogeneity within the cCAFs as reported previously. Moreover, further evaluation of gene/protein signatures from cCAFs might provide a prognostic marker for prognosing treatment response or resistance. Moreover, further understanding into their role in (m)PCa would be insightful especially for designing cCAFs targeting approaches for the treatment of mPCa. For functional analysis, current methods [57] adapted to single‐cell level e.g., single‐cell migration‐invasion assays, single‐cell collagen contraction assays, single cell proliferation assays, cancer cell‐CAF interaction assays could provide detailed insights into cCAF functions.

CAFs are thought to originate from multiple precursors. While resident tissue fibroblasts are thought to be the main cell of origin [6], studies have also reported CAFs derived from bone marrow‐derived mesenchymal stem cells [58, 59], endothelial cells [60], or epithelial cells undergoing EMT [61]. We observed two distinctly different FAP positive cell populations: CD45 positive and CD45 negative. The FAP^+^ CD45^+^ population was bigger in size, had a big nucleus, and a clearly visible cytoplasm, while the FAP^+^ CD45^−^ population was more compact. Both cell subpopulations were positive for vimentin and collagen‐I, indicating both have mesenchymal and fibroblast characteristics. Similarly, Kraman et al. [62] found a FAP^+^ CD45^−^ and a FAP^+^ CD45^+^ tumor associated stromal cell population in mouse xenograft model of lung and pancreatic cancer. They described the FAP^+^ CD45^−^ population as mesenchymal derived, collagen‐I producing CAFs, and the FAP^+^ CD45^+^ population as CD11b^+^ collagen‐I^+^ fibrocytes. Moreover, Ricci et al. [63] discovered CD45^−^ and CD45^+^ stromal subsets in the TME, the CD45^−^ subset being a CAF subpopulation, and the CD45^+^ originating from the hematopoietic stem cell (HSC) lineage. In line with these findings, we can speculate that the CD45^−^ and CD45^+^ cCAF subpopulations have different cellular origins, whereas the FAP^+^ CD45^−^ population might be derived from resident fibroblasts, or epithelial (tumor) or endothelial cells that underwent mesenchymal transition, and the FAP^+^ CD45^+^ population might be derived from bone marrow‐derived mesenchymal stem cells that are recruited towards the tumor site. However, further studies are needed to further understand the origin and clinical relevance of FAP^+^ CD45^−^ and FAP^+^ CD45^+^ cCAFs in metastatic CNPC patients. Moreover, single cell analyses as elaborated earlier could be performed on these FAP^+^ cCAF subtypes for the functional and molecular characterization including immunostainings e.g., Phalloidin to confirm the distinct morphological differences in these subtypes. Finally, the phenotype of these subtypes can be correlated with functional analysis and omics (genomic‐transcriptomic‐secretomic) using the previously reported Phenomics platform [36]. These analyses will provide us with the deeper insights into the heterogeneity of cCAFs and their role in mPCa.

Since both CTCs and cCAFs have been shown to correlate with the metastatic potential, we assessed the correlation between CTCs and cCAFs in this study. However, we found no clear correlation between CTC and cCAF counts. One possible explanation could be the distinctly different role of cCAFs and CTCs in cancer progression and cancer metastasis. While cCAFs are the cells that prepare the metastatic TME (soil) for the CTCs, CTCs are the cells that create the metastasis (seed). As such, CTC counts are prognostic markers for overall survival in castration‐resistant prostate cancer (CRPC) [31] while CAFs are associated with resistance to the treatments [16, 17, 64], which suggests that the presence of cCAFs might predict therapy response. Our cohort consisted of metastatic castration‐naïve prostate cancer (mCNPC) patients, and therefore treatment response and resistance to therapy, could not be accounted for. Another possibility for the absence of a correlation between cCAFs and CTCs could be the heterogeneity of the TME. For example, positron emission tomography (PET) scanning of prostate specific membrane antigen (PSMA) and FAP staining led to very different results. Strong PSMA positive PET staining was observed in both primary prostate cancers and lymph node metastases. However, FAP PET imaging was much more heterogeneous, found in less than half of the patients, but was more precise in the cases of reduced PSMA expression [65], suggesting FAP imaging could be useful in identifying PSMA negative/low PSMA expressing mPCa patients. More studies are needed to validate this hypothesis and clinical significance of FAP imaging, and correlation between FAP positivity (and/or FAP positive cCAFs) with tumor aggressiveness and invasiveness. Moreover, in our study, no significant correlation between cCAF counts and baseline patient clinical characteristics (initial PSA (iPSA) concentration at diagnosis, hemoglobin levels, Gleason score or M‐stage at diagnosis) was observed. However, CTC counts also did not correlate with iPSA concentration, Gleason score or M‐stage, while CTC counts have previously been reported to correlate with these clinical parameters [66]. These poor correlations could be attributed to the low number of patients included in this study. Therefore, future studies evaluating cCAF numbers in more patients and in patients at the different stages of PCa (non‐metastatic PCa, CNPC, CRPC) should be performed to underpin the clinical relevance of cCAFs in prostate cancer.

## 5 Conclusion

In this paper, we demonstrate the presence of FAP positive circulating CAFs in the blood of 18 mCNPC patients, ranging from 60 to 776 per 2 × 10^8^ mononuclear cells. Furthermore, we confirm that these cCAFs are positive for collagen‐I and vimentin. They secrete collagen‐I, which can be stimulated with TGFβ, suggesting they possess functional characteristics of CAFs. Our results further evidence the presence of two subpopulations, FAP^+^ CD45^−^ cCAFs and FAP^+^ CD45^+^ cCAFs, that are distinctly different in morphology. Future studies are warranted to explore the clinical significance of these circulating fibroblast populations.

## Conflict of interest

The authors declare no conflict of interest.

## Author contributions

RBa and LWMMT outlined and designed the study with assistance from RBo. RBa, LWMMT and JM procured the funding this project. RBa and LWMMT supervised the project. RBo performed all cCAF studies and analyzed the data, ED performed the CTC acquisition and data analysis, and KI and JK were responsible for patient inclusion. RBo, ED, KI, and JK were responsible for data acquisition and data analysis. RBo, RBa, LWMMT, JK, and JM were involved in the data interpretation. RBo prepared the figures and wrote the first drafts of the manuscript. RBa, LWMMT and JM reviewed the manuscript, and RBo and RBa revised the manuscript. All authors have read, provided their final feedback, and accepted the final manuscript.

## Peer review

The peer review history for this article is available at https://www.webofscience.com/api/gateway/wos/peer‐review/10.1002/1878‐0261.13653.

## Supporting information


**Fig. S1.** HPrFs express fibroblast‐activated protein (FAP).
**Fig. S2.** Human prostate cancer cells do not express fibroblast‐activated protein (FAP).
**Table S1.** Antibodies used for FACS.
**Table S2.** Antibodies used for immunofluorescent staining.
**Table S3.** Cell counts per individual per 2 × 10^8^ MNCs.

## Data Availability

The data that supports the findings of this study are available in Figs 1, 2, 3, 4, 5, 6 and Supporting Information of this article. This study includes no data deposited in external repositories.
