# Supplementary material for: Identification of functional and diverse circulating cancer‐associated fibroblasts in metastatic castration‐naïve prostate cancer patients
Source: Mol Oncol. 2024 Apr 17;19(7):2074–91. doi: 10.1002/1878-0261.13653 (PMC12234390; doi:10.1002/1878-0261.13653)
Supplement: Supplementary file 1 — Fig. S1. HPrFs express fibroblast‐activated protein (FAP). Fig. S2. Human prostate cancer cells do not express fibroblast‐activated protein (FAP). Table S1. Antibodies used for FACS. Table S2. Antibodies used for immunofluorescent staining. Table S3. Cell counts per individual per 2 × 10^8^ MNCs. [file MOL2-19-2074-s001.zip › Revision_Supplementary Information.docx]

**Supplementary Figures**

**
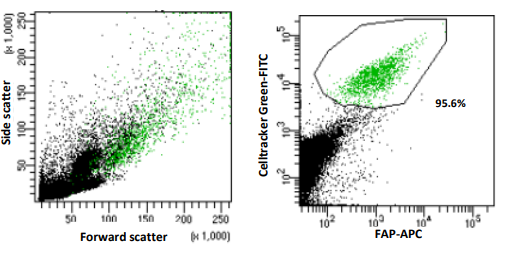
**

***Supplementary Figure 1: HPrFs express fibroblast activated protein (FAP).*** *Representative flow plots of fluorescently labeled Human Prostate Fibroblasts (HPrFs) (Celltracker Green), stained for fibroblast activated protein (FAP). FAP-negative events/cells are shown in black. 95.6 ± 2.5 % of HPrFs were FAP positive (n=3).*

***
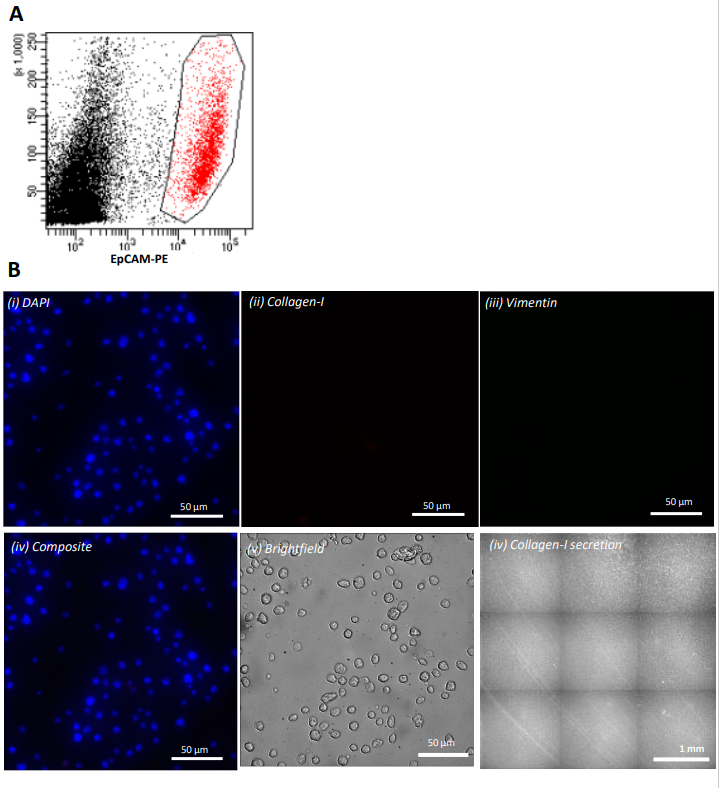
***

***Supplementary Figure 2: Human prostate cancer cells do not express fibroblast activated protein (FAP).*** *A) Representative flow plots of human prostate cancer cells (LNCaPs) spiked in mononuclear cells fraction of healthy volunteers, stained with FAP-AF647, CD45-FITC and EpCAM-PE. B) Representative images (4X, Scale bar = 50 µm, n=3) of sorted HPrFs showing (i) DAPI-stained nuclei (blue), (ii) intracellular collagen-I staining (red), (iii) intracellular vimentin staining (green) and (iv) a composite image; (v) cell morphology and density (brightfield), and (vi) collagen-I secretion captured on the PVDF membrane (scale bar = 1 mm).*

**Supplementary Table 1: Antibodies used for FACS.**

| **Antibody** | **Clone** | **RRID** | **Cat. No.** | **Source** | **Dilution** |
| --- | --- | --- | --- | --- | --- |
| FAP-AF647 | 427819 | - | FAB3715R | R&D systems | 1:50 |
| EpCAM-Pe | VU1D9 | AB_10899208 | SAB4700425 | Sigma | 1:50 |
| CD45-AF488 | 2D1 | AB_389314 | 304017 | BioLegend | 1:100 |

**Supplementary Table 2: Antibodies used for immunofluorescent staining.**

| **Primary antibody** | **RRID** | **Cat. No.** | **Source** | **Dilution** |
| --- | --- | --- | --- | --- |
| Goat anti human Collagen I | AB_2753206 | 1310-01 | Southern Bio Tech | 1:100 |
| Rabbit anti human Vimentin | AB_10695459 | 5741S | Cell Signaling | 1:100 |
| **Secondary antibody** | **RRID** | **Cat. No.** | **Source** | **Dilution** |
| Donkey anti rabbit AF488 | AB_2535792 | A21206 | Invitrogen | 1:200 |
| Donkey anti goat AF594 | AB_2534105 | A11058 | Invitrogen | 1:200 |

**Supplementary Table 3: Cell counts per individual per 2 x 10^8^ leukocytes**

|  | **Total FAP^+^** | **FAP^+^ CD45^-^** | **FAP^+^ CD45^+^** | **CTC counts** |
| --- | --- | --- | --- | --- |
|  | **mCNPC patients** | | | |
| 1 | 60 | 19 | 41 | 62 |
| 2 | 62 | 36 | 26 | 35 |
| 3 | 130 | 64 | 66 | 328 |
| 4 | 192 | 92 | 100 | 0 |
| 5 | 207 | 107 | 100 | 2 |
| 6 | 220 | 94 | 126 | 916 |
| 7 | 252 | 204 | 48 | 7 |
| 8 | 282 | 206 | 76 | 34 |
| 9 | 321 | 143 | 178 | 51 |
| 10 | 398 | 296 | 102 | 139 |
| 11 | 440 | 333 | 107 | 19 |
| 12 | 506 | 162 | 344 | 7436 |
| 13 | 528 | 297 | 231 | 167 |
| 14 | 612 | 212 | 400 | 4 |
| 15 | 628 | 264 | 364 | 1615 |
| 16 | 658 | 324 | 334 | 37 |
| 17 | 714 | 214 | 500 | 115 |
| 18 | 776 | 382 | 394 | 7 |
|  | **Healthy Donors** | | | |
| 1 | 71 | 6 | 65 | nd |
| 2 | 48 | 6 | 42 | nd |
| 3 | 21 | 5 | 16 | nd |
| 4 | 35 | 12 | 23 | nd |
| 5 | 20 | 10 | 10 | nd |
| 6 | 21 | 7 | 14 | nd |
| 7 | 56 | 0 | 56 | nd |
| 8* | 7 | 0 | 7 | nd |
| 9* | 6 | 0 | 6 | nd |
| 10* | 10 | 3 | 7 | nd |
| 11 | 0 | 0 | 0 | nd |
| 12 | 35 | 7 | 28 | nd |

*Age- and sex-matched donors; nd=not determined

** Age- and sex- matched donors*
