# Supplementary figures and images for: Identification of functional and diverse circulating cancer‐associated fibroblasts in metastatic castration‐naïve prostate cancer patients
Source: Mol Oncol. 2024 Apr 17;19(7):2074–91. doi: 10.1002/1878-0261.13653 (PMC12234390; doi:10.1002/1878-0261.13653)

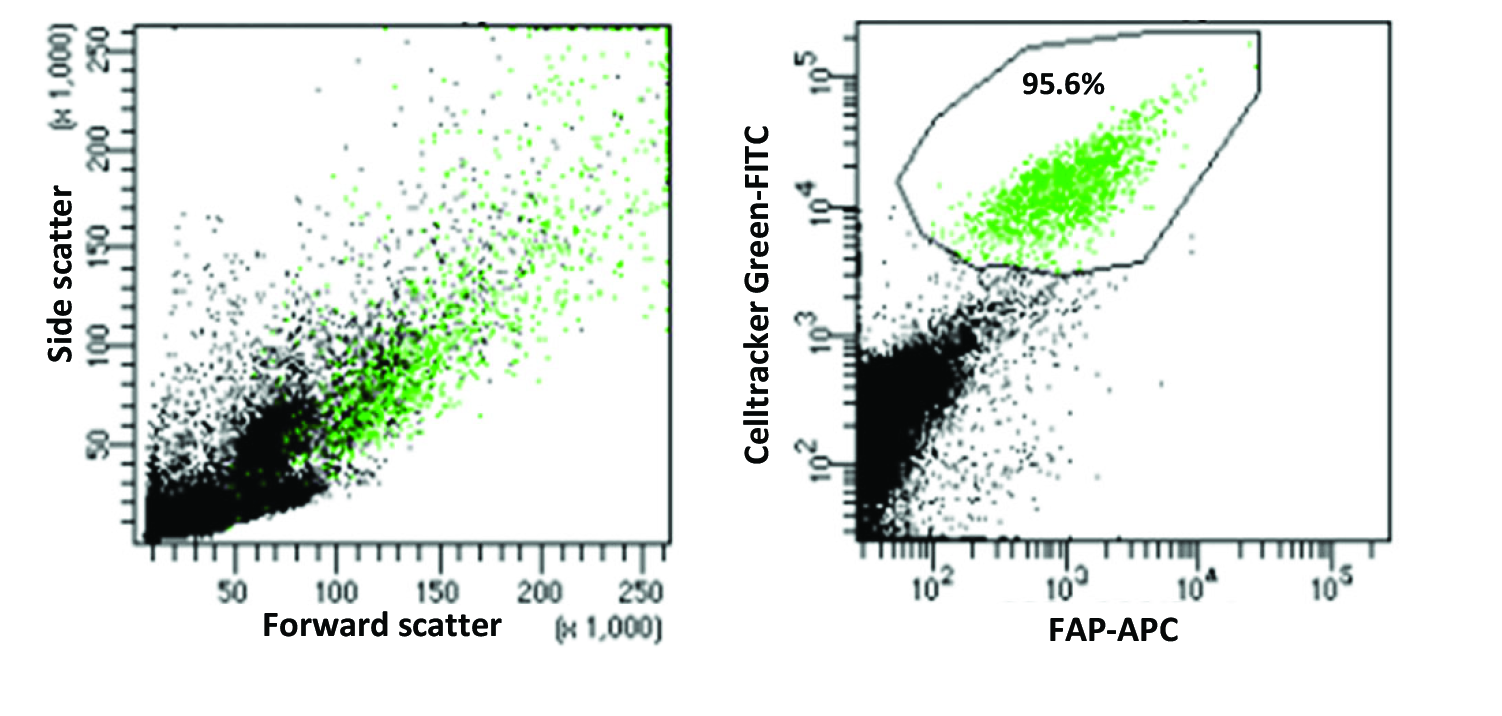

Supplement: Supplementary file 1 — Fig. S1. HPrFs express fibroblast‐activated protein (FAP). Fig. S2. Human prostate cancer cells do not express fibroblast‐activated protein (FAP). Table S1. Antibodies used for FACS. Table S2. Antibodies used for immunofluorescent staining. Table S3. Cell counts per individual per 2 × 10^8^ MNCs. [file MOL2-19-2074-s001.zip › Revised_Supplementary Figures_Circulating fibroblasts_Richell-1.tif]

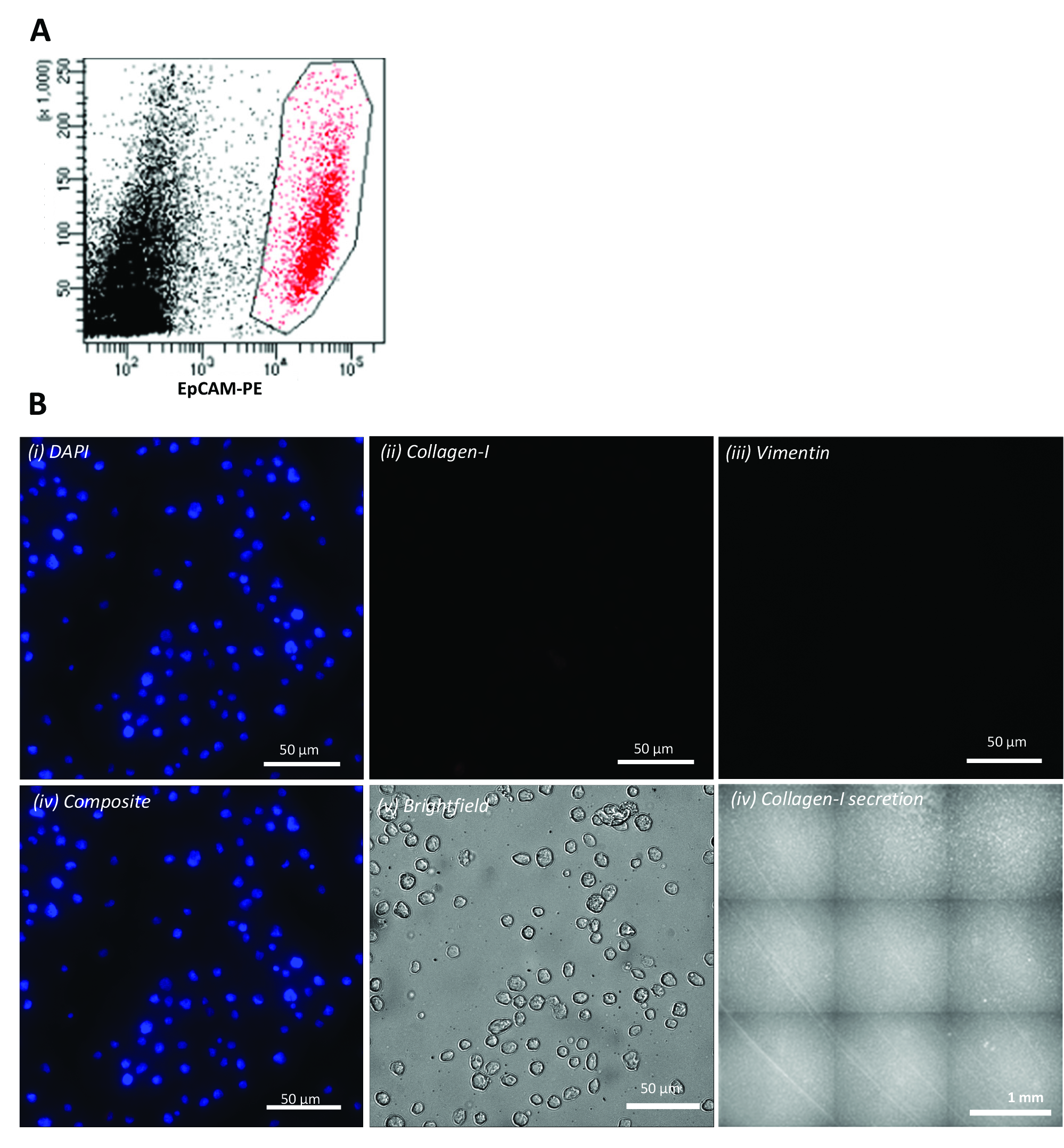

Supplement: Supplementary file 1 — Fig. S1. HPrFs express fibroblast‐activated protein (FAP). Fig. S2. Human prostate cancer cells do not express fibroblast‐activated protein (FAP). Table S1. Antibodies used for FACS. Table S2. Antibodies used for immunofluorescent staining. Table S3. Cell counts per individual per 2 × 10^8^ MNCs. [file MOL2-19-2074-s001.zip › Revised_Supplementary Figures_Circulating fibroblasts_Richell-2.tif]
